# Clinical and genetic analysis of lipoprotein glomerulopathy patients caused by *APOE* mutations

**DOI:** 10.1002/mgg3.1281

**Published:** 2020-05-22

**Authors:** Mingxin Yang, Qinjie Weng, Xiaoxia Pan, Hafiz Muhammad Jafar Hussain, Shuwen Yu, Jing Xu, Xialian Yu, Yunzi Liu, Yuanmeng Jin, Chunli Zhang, Xiao Li, Hong Ren, Nan Chen, Jingyuan Xie

**Affiliations:** ^1^ Department of Nephrology Institute of Nephrology Ruijin Hospital, Shanghai Jiao Tong University School of Medicine Shanghai China

**Keywords:** *APOE*, lipoprotein glomerulopathy, mutation, proteinuria

## Abstract

**Background:**

Lipoprotein glomerulopathy (LPG) is a rare kidney disease caused by *APOE* mutations. The aim of this study was to correlate the genetic and clinical features of LPG.

**Methods:**

Totally eight LPG patients were recruited in this study and Sanger sequencing of *APOE* was performed for all available family members. Clinical and histological features were analyzed. A literature review of LPG was also conducted.

**Results:**

Genetic analysis revealed five patients with *APOE‐*Kyoto, two with *APOE‐*Osaka/Kurashiki, and one with *APOE‐*Chicago mutations. LPG patients with urine protein reduced more than 50% had a slower decrease in renal function than those with less urine protein reduction (estimated glomerular filtration rate reduction rate −5.0 ± 0.8 vs. 1.5 ± 0.7 ml/min per 1.73 m^2^⋅month^−1^, *p* = .03). We then enrolled 95 LPG patients from previous studies and this study. LPG patients had higher blood pressure (mean arterial pressure: 109.4 ± 19.4 vs. 94.4 ± 11.1 mmHg, *p* < .001) than the control group. Interestingly, patients with *APOE* mutations in the LDL receptor binding region had higher serum apolipoprotein E (apoE) levels [ln(apoE): 2.7 ± 0.4 vs. 2.0 ± 0.5 mg/dl, *p* < .001] in comparison to other domains.

**Conclusion:**

Here, we report for the first time *APOE*‐Osaka/Kurashiki and *APOE*‐Chicago mutations in the Chinese population. LPG was associated with higher blood pressure and serum apoE levels were higher in patients with mutations in LDL receptor binding region. In addition, the findings further indicated that treatment of proteinuria might slow down renal function progression in these patients.

## Graphical abstract

For the first time *APOE*‐Osaka/Kurashiki and *APOE*‐Chicago mutations reported in Chinese; Lipoprotein glomerulopathy (LPG) patients with urine protein remission had slower renal function decrease; LPG was associated with higher blood pressure; Serum apoE levels were higher in patients with low‐density lipoprotein receptor binding region mutations of APOE

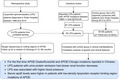

## 1 INTRODUCTION

Lipoprotein glomerulopathy (LPG, MIM #611771) is a rare renal disorder and characterized by renal lipidosis, renal insufficiency, and proteinuria. The Japanese scholar Saito described LPG for the first time in 1989 (Saito et al., 1989). To the best of our knowledge, approximately 200 cases of LPG have been reported worldwide so far and most of them were from East Asia presenting with founder effect along with several cases from Americas and Europe (Batal, Fakhoury, Groopman, D'Agati, & Morris, 2019; Magistroni et al., 2013; Rovin et al., 2007; Russi et al., 2009; Saito & Matsunaga, 2014). Most LPG patients had mutations in *APOE* (apolipoprotein E, MIM* 107741), except for one reported by Zhang et al. (2008

). The mRNA of *APOE* is translated to a protein of 317 amino acids and the mature protein loses its signal peptide of the first 18 amino acids (Marais, 2019). Here we mainly use the numbering applicable to the mature protein as in previous publications, while only in Sanger sequencing results we use the numbering of 317 amino acids (NP_000032.1) for location. APOE is a ligand for the low‐density lipoprotein (LDL) receptor, heparan sulphate proteoglycan (HSPG) and LDL receptor‐related protein and it includes an LDL receptor binding region (amino acids 136–150) and a HSPG binding region (amino acids 142–147) in the N‐terminal domain. APOE has three variants ε2, ε3, and ε4, depending on the different amino acids at positions 112 and 158, and the APOE ε3 is regarded as the wild type (Bennet et al., 2007). Till date, nearly 70 mutations have been identified in *APOE* associated with LPG, dysbetalipoproteinemia, hypertriglyceridemia and Alzheimer's disease, etc. The first mutation associated with LPG in *APOE* was *APOE*‐Sendai reported in Japan (Saito et al., 1989). More than 10 types of LPG‐related mutations have been identified in *APOE* showing autosomal dominant inheritance patterns with incomplete penetrance (Matsunaga & Saito, 2014). LPG‐related mutations found in China include *APOE*‐Kyoto, *APOE*‐Tokyo/Maebashi, *APOE*‐Guangzhou, and *APOE*‐Chengdu (Han et al., 2010; Wu, Yang, & Hu, 2018). Hu et al. (2014) found dozens of *APOE*‐Kyoto mutant LPG patients in Sichuan Province of China, which was the largest group of LPG patients reported so far (Hu et al., 2014).

Clinical manifestations of LPG patients include mild nephrotic problem initially but later progress to nephrotic syndrome with elevated levels of serum apolipoprotein E (apoE), hyperlipidemia, hypertension and microscopic hematuria (Hu et al., 2014; Saito & Matsunaga, 2014). The abnormal plasma lipoprotein profile of patients resembles type III hyperlipoproteinemia, while the defect is limited to the kidney. The systemic manifestations of type III hyperlipidemia, such as xanthoma, are hardly seen in patients with LPG (Matsunaga & Saito, 2014). The diagnosis of LPG mainly relies on histopathology. In light microscope, pale‐stained thrombus‐like substances can be seen in the glomerular capillary lumen. Using snap‐frozen kidney sections, immunofluorescence shows that apolipoprotein B (apoB), apoE and Sudan III staining are positive (Hu et al., 2014). Fibrate lipid‐lowering drug therapy is used to cure this disease. However, few studies have dealt with the relation between genotypes and phenotypes. Here, we present the clinical features of eight Chinese patients with LPG in our department recruited in the last 20 years (1998–2019), along with 87 patients from already published literature to explain the association between genotype–phenotype correlation.

## 2 MATERIALS AND METHODS

### 2.1 Ethical compliance

The study was approved by the Institutional Review Board of Ruijin Hospital, Shanghai Jiao Tong University School of Medicine and was conducted in accordance with the principle of the Helsinki Declaration.

### 2.2 Study design

A total of eight biopsy‐proven LPG patients whose DNA samples available were enrolled. The patients were diagnosed in Ruijin Hospital between 1998 and 2019. Written informed consent was obtained from all participants. Detailed clinical baseline data including age, gender, mean arterial pressure (MAP), albumin (Alb), hemoglobin (Hb), urine protein (Upro), serum creatinine and estimated glomerular filtration rate (eGFR), serum triglyceride (TG), serum total cholesterol (TC), and serum apoE were collected from all patients at the time of kidney biopsies. eGFR was calculated using the chronic kidney disease (CKD) Epidemiology Collaboration equation (EPI‐eGFR; Levey et al., 2009).

### 2.3 Sanger sequencing

DNA samples of the patients and their family members were extracted from peripheral blood leukocytes or from epithelial cells in saliva by using QuickGene DNA Whole Blood kit L (FujiFilm Life Sciences). We designed primers (Table S1) covering the four exon regions of *APOE*, and the reference gene sequence was derived from the NCBI database (GenBank accession no. NM_000041.4). After polymerase chain reaction (PCR) amplification, Sanger sequencing and analysis were performed.

### 2.4 Literature review

We sought studies published between 1 January 1989, when LPG was first reported (Saito et al., 1989), and 30 September 2018 on LPG patients with *APOE* mutations. Electronic searches, limited to English/Chinese language, were performed using MEDLINE, EMBASE, Wanfang Data (Chinese), and the Chinese National Knowledge Infrastructure Database (Chinese) with the keywords “lipoprotein glomerulopathy”. A flow chart is shown in Figure S1. We retrieved 28 articles from 327 records, limited to reports of LPG patients with *APOE* mutations. From the selected papers, we enrolled 87 patients totally, of whom 52 patients’ individual baseline data were available.

We also enrolled non‐LPG nephrotic syndrome (NS) patients as a control group. Inclusion criteria were primary focal segmental glomerulosclerosis (FSGS)/minimal change disease (MCD) defined by kidney biopsy and NS defined by Upro > 3.5 g/24 hr and plasma‐albumin < 30 g/L. Exclusion criteria were eGFR < 15 ml/min per 1.73 m^2^ at the time of biopsy or any evidence of systemic lupus erythematosus, renal amyloidosis, obesity‐related glomerulopathy, diabetic nephropathy, systemic vasculitis, human immunodeficiency virus (HIV)‐associated nephropathy, or other systemic diseases. As a result, 60 patients with NS (27 FSGS and 33 MCD) from our department during 2013–2015 were enrolled. A comparison with the control group in clinical manifestations and a correlation analysis of genetic and clinical features was performed.

### 2.5 Statistical analysis

The continuous variables conformed to the normal distribution are presented as means ± *SD*, and independent *t*‐test was employed to evaluate the difference between the two groups. Continuous variables were transformed to a logarithmic scale when they did not pass the normality test. The categorical variables are expressed in terms of frequency (percentage) using the Pearson chi‐square test. The data from published literature were processed using an established statistical method (Altman, Machin, Bryant, & Gardner, 2000). Statistical analyses were performed using SPSS 19.0 software and statistical charts were drawn by GraphPad Prism 7 software. Differences were considered statistically significant with a two‐side *p* < .05.

## 3 RESULTS

### 3.1 Demographic data and clinico‐histological features

A total of eight patients including six males and two females, aged 16–50 years old, from unrelated Han families were recruited in this study. They came from different provinces of China including Jiangxi, Zhejiang, Hubei, Xinjiang, Anhui, and Guizhou. Interestingly family history was found in five cases.

Baseline clinical characteristics are shown in Table 1. All the patients presented with moderate to nephrotic range proteinuria (range, 1.5–10.5 g/24 hr). Systemic manifestations of lipidoses were not found. Average eGFR of patients was 83.6 ± 20.2 ml/min per 1.73 m^2^ (range of 51.9–110.8 ml/min per 1.73 m^2^) at the time of kidney biopsy, while eGFRs of two patients were less than 60 ml/min per 1.73 m^2^. Elevated TG levels (higher than 1.7 mmol/L) were observed in seven of the eight patients and more than half of the patients presented with elevated TC levels (higher than 5.7 mmol/L). Serum apoE was tested in patients and only two of them presented with elevated apoE levels (higher than 5.3 mg/dl).

**TABLE 1:** Baseline clinical characteristics of lipoprotein glomerulopathy patients

Parameters	Subjects							
	F171101‐II4	F17A0528‐II2	F15A1307‐II1	F15A1558‐II1	F98034‐III1	F16A0702‐II2	F16A1578‐II1	F19A0312‐II4
Age (year)	42	16	30	50	38	22	28	50
Gender	M	M	F	M	F	M	M	M
Family history	Y	Y	Y	N	Y	Y	N	N
MAP (mmHg)	91	116	84	117	117	141	141	106
Alb (g/L)	33.6	20.0	34.0	20.0	22.3	21.0	19.1	31.2
Hb (g/L)	121	106	117	137	101	115	117	NA
Upro (g/24 hr)	1.5	5.1	1.5	5.0	10.5	10.0	8.8	5.7
eGFR (ml/min per 1.73 m^2^)	56.2	110.8	101.2	84.5	93.4	98.5	71.9	51.9
TG (mmol/L)	3.0	2.7	1.5	3.0	3.7	1.9	2.1	4.3
TC (mmol/L)	7.6	6.4	3.4	7.8	5.8	3.5	9.9	5.2
apoE (mg/dl)	4.5	6.4	3.6	6.6	NA	3.6	4.1	NA
*APOE* mutation	Kyoto	Kyoto	Kyoto	Kyoto	Kyoto	Osaka	Osaka	Chicago
*APOE* genotype	ε3/ε3	ε3/ε4	ε3/ε3	ε3/ε4	ε3/ε3	ε3/ε3	ε3/ε4	ε3/ε3
Pathology^a^								
Global sclerosis	3	1	1	1	0	2	2	0
Segmental sclerosis	0	0	0	0	0	1	1	0
Interstitial fibrosis	2	1	0	1	1	1	2	1
Interstitial inflammatory cell infiltration	2	1	0	1	0	1	2	1
Tubular atrophy	2	1	0	1	1	1	2	0
Vascular lesion	1	1	0	1	0	0	1	1

Note

^a^ The standards of pathology grade were as follows:

Global sclerosis was defined as sclerosis involving the entire glomerular tuft. Global sclerosis was scored by the percentage of glomeruli with these lesions: 0, 0%; 1, <10%; 2, 10%–24%; 3, ≥25%.

Segmental sclerosis was defined as tufts involved with sclerosis other than global sclerosis. Segmental sclerosis was scored as follows: 0, absent; 1, present.

The severity of interstitial fibrosis, interstitial inflammatory cell infiltration and tubular atrophy are defined as follows: 0, 0% area involved; 1, <10%; 2, 10%–24%; 3, ≥25%.

The vascular lesion was defined by arterial hyaline change and vascular wall thickening: for either, definitions were 0, absent, or 1, present.

Abbreviations: Alb, albumin; apoE, apolipoprotein E; Chicago, *APOE*‐Chicago (p.R147P); EPI‐eGFR, estimated glomerular filtration rate using EPI; F, female; Hb, hemoglobin; Kyoto, *APOE*‐Kyoto (p.R25C); M, male; MAP, mean arterial pressure; N, No; NA, not available. All the parameters in this table were obtained at time of kidney biopsy; Osaka, *APOE*‐Osaka/Kurashiki (p.R158P); TC, total cholesterol; TG, triglyceride; Upro, urine protein; Y, Yes.

**FIGURE 1:**
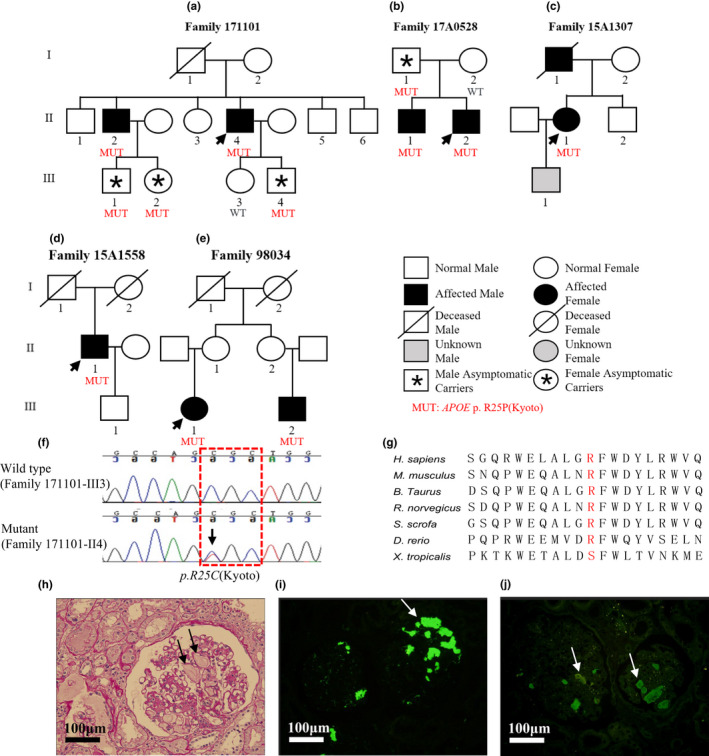
Family pedigrees, genomic sequencing, and kidney biopsy specimens of patients with *APOE*‐Kyoto mutation. (a–e) Family pedigrees of five lipoprotein glomerulopathy (LPG) families with *APOE*‐Kyoto mutation. Arrows show the probands. (f) Representative chromatograms of control (upper) and mutant (lower). The position of *APOE*‐Kyoto mutation is marked by arrow and red dashed box shows the genetic code of arginine (Arg). (g) Multiple sequence alignment of different species shows a high level of conservation at the position of substitution. (h) A representative glomerulus from patient F171101‐II4 shows marked dilatation of the capillary lumen in the glomeruli by a pale‐stained thrombus‐like substance (periodic acid‐Schiff stain). Immunofluorescence shows that apoE (i) and apoB (j) are present primarily in the capillary lumen. GenBank accession no. NM_000041.4. Scale bars 100 μm

**FIGURE 2:**
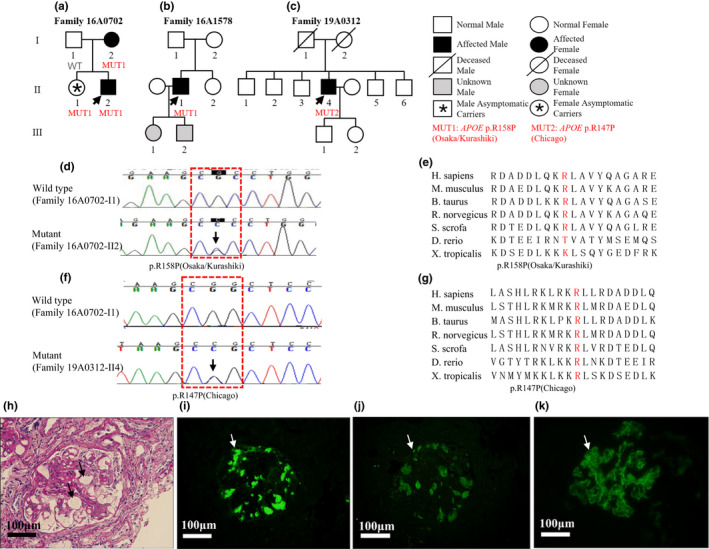
Family pedigrees, genomic sequencing, and kidney biopsy specimens of patients with *APO* *E*‐Osaka/Kurashiki and *APOE*‐Chicago mutations. Family pedigrees of LPG patients with *APOE*‐Osaka/Kurashiki mutation (a, b) and *APOE*‐Chicago mutation (c). Arrows show the probands. (d, f) Representative chromatograms of control (upper) and mutant (lower) for both mutations. The positions of the Osaka/Kurashiki mutation (D)/Chicago mutation (f) are marked by arrow and red dashed boxes show the genetic code of arginine. (e, g) Multiple sequence alignment of different species shows that the substituted amino acids are highly conserved. (h) A representative glomerulus from patient F16A1578‐II1 shows marked dilatation of the capillary lumen in the glomeruli by a pale‐stained thrombus‐like substance (periodic acid‐Schiff stain). Immunofluorescence shows that apoE (i) and apoB (j) are present primarily in the capillary lumen. (k) Deposition of immunoglobulin A (IgA) was found in the glomerulus of patient F16A1578‐II1. GenBank accession no. NM_000041.4. Scale bars 100 μm

Light microscopy of the renal tissues revealed the expansion of the capillary lumen. Furthermore, immunofluorescence microscopy showed deposition of apoE and apoB. Deposition of the immunoglobulin A (IgA) antibody was found in the glomerulus of one patient (F16A1578‐II1; Figures 1h–j and 2h–k).

### 3.2 Genotyping

Sequencing of the entire *APOE* coding region revealed that five patients (F171101‐II4, F17A0528‐II2, F15A1307‐II1, F15A1558‐II1, and F98034‐III1) had heterozygous *APOE‐*Kyoto mutation (chr19‐45411100, c.127C>T, p.R43C) which could substitute the amino acid at position 25 from arginine (Arg) to cysteine (Cys). Three of them had an *APOE* genotype of ε3/ε3 and two ε3/ε4. Additionally, two patients (F16A0702‐II2 and F16A1578‐II1) had heterozygous *APOE‐*Osaka/Kurashiki mutation (chr19‐45412080, c.527G>C, p.R176P) which could substitute the amino acid at position 158 from Arg to proline (Pro). They had genotypes of ε3/ε3 and ε3/ε4 respectively. Moreover, one patient (F19A0312‐II4) was detected with heterozygous *APOE‐*Chicago mutation (chr19‐45412047, c.494G>C, p.R165P) which could replace Arg by Pro in position 147 at protein level, with a genotype of ε3/ε3. Furthermore, Sanger sequencing was performed for F171101‐II4, F17A0528‐II2, F98034‐III1 and F16A0702‐II2 families to validate the inheritance pattern. Interestingly, we found asymptomatic carriers in three families (F171101, F17A0528, and F16A0702), and the penetrance was 54.5% (six patients and five asymptomatic carriers of the three families; Figures 1a,b and 2a).

### 3.3 Treatment and follow‐up

Patients were treated with statins (F15A1307‐II1, F15A1558‐II1, F16A0702‐II2, and F16A1578‐II1) or fibrates (F171101‐II4, F17A0528‐II2, F15A1558‐II1, F16A0702‐II2, F16A1578‐II1, and F19A0312‐II4) and angiotensin receptor blockers (ARBs; all of them). Two of them (F17A0528‐II2 and F16A0702‐II2) went through plasma exchange or blood lipid adsorption and their Upro and serum lipid levels went down for a while. But shortly after the treatment, the values returned to baseline. One patient (F15A1558‐II1) received glucocorticoid treatment for more than three years and his Upro remained 2.6–5.0 g/24 hr. One (F98034‐III1) of the six patients was lost to follow‐up, and the interval of patient F19A0312‐II4 from renal biopsy to the data collection was too short. The remaining patients were followed‐up for a median of 22 (range of 12–36) months. Patient F171101‐II4 stopped fenofibrate therapy because of temporarily elevated serum creatinine or liver enzyme levels. His proteinuria once decreased to 0.4 g/24 hr but increased after he ceased the treatment, as well as his apoE level. Patient F16A1578‐II1 was treated with statins instead of fibrates due to high TC level. We screened family members of F16A0702 (Figure 2a) at their first hospital visit and found that F16A0702‐I2 had normal Upro level at that time. One year later, when we validated that she had the *APOE*‐Osaka/Kurashiki mutation by Sanger sequencing, and her Upro had reached 2.1 g/24 hr.

**FIGURE 3:**
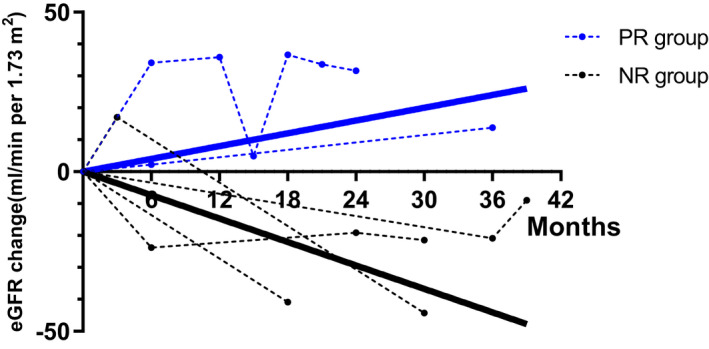
Estimated glomerular filtration rate (eGFR) trends in urine protein partial remission (PR) patients (blue) and nonremission (NR) patients (black). eGFR changes in each patient are presented with dashed line. The lines with mean eGFR change linear regression slope of PR patients (blue continuous line) and NR patients (black continuous line) show better kidney prognosis of urine protein PR patients. PR (partial remission) group: urine protein at the 12th month of follow‐up decreased equal to or more than 50% compared to baseline; NR (none remission) group: urine protein at the 12th month of follow‐up decreased less than 50% compared to baseline.

We divided the patients into PR (partial remission) group (*n* = 2) and NR (nonremission; *n* = 4) groups based on whether their Upro at the 12th month of follow‐up decreased equal to or more than 50% compared to baseline. Interestingly, the speed of eGFR decline in patients from the PR group was slower (1.5 ± 0.7 vs. −5.0 ± 0.8 ml/min per 1.73 m^2^⋅month, *p* = .03; Figure 3).

### 3.4 Literature review

Furthermore, we did a literature review of already reported LPG patients having *APOE* mutations. By inclusion and exclusion criteria, we included 87 LPG patients and they all had *APOE* mutations. Hence, our study included a total of 95 patients from previous reports and from our department to correlate the genetic and clinical features of LPG patients.

**FIGURE 4:**
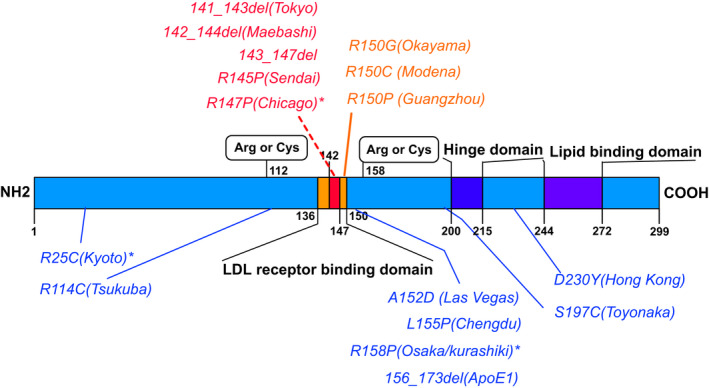
*APOE* mutations in different regions. *APOE* mutation frequency causing lipoprotein glomerulopathy (LPG) is higher in the low‐density lipoprotein (LDL) receptor binding region (amino acids 136–150) or around it, especially in the heparan sulphate proteoglycan (HSPG) binding region (amino acids 142 to 147). Mutations in the HSPG binding region (*APOE* Tokyo/Maebashi/Sendai/Chicago) are in red font. Mutations in the LDL receptor binding region outsides the HSPG binding region (*APOE* Okayama/Modena/Guangzhou) are in yellow font. Mutations outside the LDL receptor binding region (*APOE* Kyoto/Tsukuba/Las Vegas/Chengdu/Osaka/Kurashiki/ApoE1/Hongkong/Toyonaka) are in blue font

Most LPG‐related mutations are in or around the LDL receptor binding region of APOE, indicating the mechanistic insight for their roles in the pathogenesis of LPG (Figure 4). Thus, we divided the patients into groups depending on the mutation sites (in or outside of the LDL receptor and HSPG binding region).

There were 36 patients with mutations in LDL receptor binding region (amino acids 136–150) of whom 31 patients with mutations in HSPG binding region (amino acids 142–146), and 59 patients with mutations in other domains of APOE protein (Table 2). It shows that the LDL receptor binding region is a hot spot site for genetic alterations. Furthermore, patients with LPG had higher blood pressure than the control group (MATERIALS AND METHODS 2.4) (MAP: 109.4 ± 19.4 vs. 94.4 ± 11.1 mmHg, *p* < .001), while Upro [ln (Upro): 1.3 ± 0.9 vs. 1.9 ± 0.5 g/24 hr, *p* < .001) and TC levels [ln (TC): 1.8 ± 0.5 vs. 2.3 ± 0.4 mmol/L, *p* < .001] were lower. Interestingly, patients with *APOE* mutations in the LDL receptor binding region had higher serum apoE levels than mutations in other regions [ln(apoE): 2.7 ± 0.4 vs. 2.0 ± 0.5 mg/dl, *p* < .001]. Among patients with mutations in the LDL receptor binding region, patients with mutations in the HSPG binding region consisted of the majority (*N* = 31 [86%]). Among patients with LDL receptor binding region mutations in or outside of the HSPG binding region, the comparison of the clinical characters showed that patients with mutations in the HSPG binding region had higher serum apoE levels than the others (Table 2). Meanwhile, although not statistically significant, compared with patients having mutations in the LDL receptor binding region and outside of the HSPG binding region, patients with *APOE* mutations in the HSPG binding region had a trend of younger onset age (31.3 ± 17.7 vs .40.0 ± 18.7, *p* = .32), higher Upro [ln(Upro): 1.0 ± 0.9 vs. 0.3 ± 0.6 g/24 hr, *p* = .3], and higher TG [ln(TG): 1.1 ± 0.5 vs. 0.6 ± 0.5 mmol/L, *p* = .06].

**TABLE 2:** Distribution of *APOE* mutations in lipoprotein glomerulopathy patients

	Mutation location			
	LDLR‐HSPG	LDLR‐non‐HSPG	Other regions	Control
*n*	31	5	59	60
Male, *n* (%)	15 (48.4)	1 (20.0)	31 (52.5)	31 (51.7)
Positive family history, *n* (%)	14 (45.2)	3 (60.0)	12 (32.4)	—
Age (year)	31.3 ± 17.7	40.0 ± 18.7	37.0 ± 12.9	35.0 ± 12.8
MAP (mmHg)	105.4 ± 14.0^a^	107.2 ± 25.1	111.0 ± 20.8^a^	94.4 ± 11.1
ln(Upro) (g/24 hr)	1.0 ± 0.9^a^, ^b^	0.3 ± 0.6^a^	1.5 ± 0.9^a^	1.9 ± 0.5
eGFR (ml/min per 1.73 m^2^)	95.4 ± 35.3	104.4 ± 28.6	83.9 ± 31.7^a^	95.3 ± 27.5
ln(TG) (mmol/L)	1.1 ± 0.5	0.6 ± 0.5	1.1 ± 0.5	1.1 ± 0.6
ln(TC) (mmol/L)	1.8 ± 0.4^a^	1.9 ± 0.3^a^	1.8 ± 0.5^a^	2.3 ± 0.4
ln(apoE) (mg/dL)^c^	2.7 ± 0.4^a^, ^b^	1.9 ± 0.3	2.0 ± 0.5^a^	1.8 ± 0.3

Abbreviations: apoE, apolipoprotein E; EPI‐eGFR, estimated glomerular filtration rate using EPI; LDLR‐HSPG, mutations in HSPG binding region within low‐density lipoprotein receptor binding region; LDLR‐nonHSPG, mutations in low‐density lipoprotein receptor binding region outsides HSPG binding region; MAP, mean arterial pressure; *n*, number; TC, total cholesterol; TG, triglyceride; Upro, urine protein.

^a^ Compared with control group (MATERIALS AND METHODS 2.4), *p* < .05.

^b^ Compared with other regions group, *p* < .05.

^c^ ln(apoE): 83 of the 95 LPG patients and 19 (two focal segmental glomerulosclerosis and 17 minimal change disease) of the control group had apoE data. Among the 83 LPG patients, 27 had mutations in LDLR‐HSPG, two in LDLR‐nonHSPG, and 54 in other regions.

Furthermore, 48 LPG patients with serum apoE available were classified into two groups according to the median serum apoE level (11.2 mg/dl). Patients in the high apoE level group had higher TC levels than those in the low apoE group (ln(TC): 1.9 ± 0.4 vs. 1.5 ± 0.5 mmol/L, *p* = .006; Table S2). Furthermore, although not statistically significant, patients in the high apoE group showed a trend of younger onset age (31.1 ± 19.1 vs. 34.9 ± 14.0 years old, *p* = .4), higher MAP (106.8 ± 15.1 vs. 101.7 ± 15.6 mmHg, *p* = .4), higher Upro [ln(Upro): 1.0 ± 0.9 vs. 0.8 ± 1.2 g/24 hr, *p* = .5], and higher TG levels [ln(TG): 1.1 ± 0.5 vs. 0.9 ± 0.6 mmol/L, *p* = .2] compared with patients in the low apoE group.

To further investigate the relationship between LPG and MAP, we built a linear regression model. Compared with the control group, patients in the LPG group were associated with a higher MAP before adjusted (*β* = 10.2 [4.9–15.6], *p* < .001), after adjusted for age and sex in model II (*β* = 10.7 [5.7–15.7], *p* < .001), and after adjusted for age, sex, eGFR, Upro, ln(TG) and ln(TC) in model III (*β* = 11.6 [5.6–17.6], *p* < .001; Table 3). These results suggested that LPG was a risk factor for hypertension independent of other common factors.

**TABLE 3:** Relationship between lipoprotein glomerulopathy and mean arterial pressure in different models

Variable	Model I		Model II		Model III	
	β (95% CI)	*p*‐value	β (95% CI)	*p*‐value	β (95% CI)	*p*‐value
Control^a^	Ref		Ref		Ref	
LPG	10.2 (4.9–15.6)	<.001	10.7 (5.7–15.7)	<.001	11.6 (5.6–17.6)	<.001

Note

Model I, unadjusted; Model II, adjusted for age and sex; Model III, adjusted for age, sex, Estimated glomerular filtration rate, ln (urine protein), ln (triglyceride), ln (total cholesterol).

Abbreviations: LPG, lipoprotein glomerulopathy; CI, confidence interval; Ref, reference.

^a^ The nephrotic syndrome control group (MATERIALS AND METHODS 2.4).

## 4 DISCUSSION

Lipoprotein glomerulopathy is a rare inherited kidney disease and most patients were reported in East Asia. Although LPG is one of apoE related lipoprotein disorders, it affects predominantly in kidney and the histological features are dilated glomerular capillaries with lipoprotein thrombi. By genetic testing, several pathogenic *APOE* mutations of LPG have been reported. Most of the mutations are located close to LDL receptor binding site, however, mutations far from the LDL receptor binding site have also been reported. It is interesting to know whether different types of mutations or mutations in different domains are associated with the phenotypes of LPG. In our study, eight probands diagnosed with LPG were enrolled in Ruijin Hospital. By Sanger sequencing of *APOE*, we found three *APOE* mutations including *APOE*‐Kyoto (R25C), *APOE*‐Osaka/Kurashiki (R158P) and *APOE*‐Chicago (R147P). For the first time we report *APOE*‐Osaka/Kurashiki outside of Japan (Saito & Matsunaga, 2011). *APOE*‐Chicago was also first reported in Chinese LPG patients which was first reported in a male Mexican‐American patient (Sam et al., 2006) and later in a Japanese female patient (Kodera et al., 2017).

According to an extended study of patients with LPG (Hu et al., 2014), 27.7% LPG patients had a positive family history and the penetrance of *APOE* mutations was 55.6%. So our study validated the incomplete penetrance of LPG‐related *APOE* mutations as a similar value of 54.5%. These findings indicated that additional factors were involved in the induction of LPG such as the Fcg receptor (FcRg) deficiency (Ito et al., 2012; Kanamaru et al., 2002; Miyahara et al., 2012). Interestingly, we also found that higher blood pressure was associated with LPG compared to patients with nephrotic syndrome independent to other factors like age, sex, eGFR, Upro, TC, and TG. Studies also reported such phenomenon (Hu et al., 2014). One possible explanation might be that hypertension is due to endothelium injury caused by lipoprotein thrombi in glomerular capillary.

In addition, we found that patients with *APOE* mutations in the binding region of HSPG (within the LDL receptor binding region) had higher serum apoE levels. Among patients with LDL receptor binding site mutations, most of their mutations were in the binding region of HSPG. Patients with *APOE* mutations in the HSPG binding region had a trend of younger onset age, higher Upro, and higher TG, although not statistically significant. As serum apoE plays its role in lipid metabolism through the LDL receptor, the HSPG and LDL receptor‐related protein (LRP) pathway (Mahley & Rall Jr., 2000), mutations could alter the configuration of the APOE protein and further impact its binding ability to LDL receptor, which could explain the formation of lipoprotein thrombi in the glomerulus.

Only three mutations (*APOE* Okayama/Modena/Guangzhou) were inside the LDL receptor binding region and outside the binding region of HSPG, and they were all at position 150 at the protein level. We found that both *APOE‐*Okayama (R150G; Kinomura et al., 2008) and *APOE‐*Modena (R150C; Magistroni et al., 2013; Russi et al., 2009) were located on the ε2 allele, while *APOE* allele of *APOE‐*Guangzhou (R150P) was not reported (Luo et al., 2008). Almost all other mutations were located on the ε3 allele except for a new reported mutation *APOE‐*Toyonaka (Fukunaga et al., 2018; Hirashima et al., 2018). This interesting phenomenon may be explained by this reason: as is reported in APOE2 (variant ε2), due to the cysteine 158 substitution, aspartic acid 154 changes its ionic interaction with arginine 150 instead of arginine 158, disrupting LDL receptor binding significantly (Marais, 2019). These results indicate that the three mutations change the APOE three‐dimensional structure and lead to the disease. This finding is consistent with reports that mutations on APOE3 (variant ε3) of LPG induce thermodynamic destabilization and enhanced aggregation (Georgiadou et al., 2013; Katsarou, Stratikos, & Chroni, 2018). On the other hand, LPG is a heterogenous disease in pathology, especially the recently discovered mutation *APOE*‐Toyonaka with membranous nephropathy‐like kidney pathology (Fukunaga et al., 2018; Hirashima et al., 2018), suggesting that genetic factor is significant in LPG (Saito et al., 2020).

Though no effective treatment has been established, fibrates and ACEI/ARB may be effective for LPG remission (Arai et al., 2003; Ieiri, Hotta, & Taguma, 2003; Matsunaga et al., 2009). Different therapy effects of lipid‐lowering drugs and ACEI/ARB used in all the eight LPG patients indicated heterogeneity of the disease. Meanwhile, plasma exchange or blood lipid adsorption was performed in two patients and their Upro and serum lipid levels relieved only for a short time, so the effect of this treatment was uncertain as reported (Li et al., 2014; , Oikawa, Sato, & Sasaki, 1999). Furthermore, one patient received glucocorticoid treatment for more than three years and appeared to have steroid‐dependent nephrotic syndrome. Further work is required in consideration of the mutations and heterogeneity of each patient. At the same time, we found that patients in the proteinuria PR group had a slower renal function deterioration rate compared to that in NR group. Our results indicate that Upro during follow‐up might be an alternative marker for renal function deterioration in LPG patients, and further suggests that urine protein decreasing therapy in LPG patients might be crucial for delaying the progression of renal failure. There are some limitations in our study. Firstly, it is a small sample size study which limited the power to detect more correlations between genotypes and phenotypes. Second, we only performed Sanger sequencing on *APOE*, which may have lead lack pathogenetic mutations on other unknown genes. Finally, we did not carry out functional studies to reveal the mechanisms of these mutations.

Our study showed the essentiality of genetic testing for LPG. We associated *APOE*‐Osaka/Kurashiki (R158P) and *APOE*‐Chicago (R147P) in Chinese LPG patients for the first time. We found that LPG patients with urine protein remission had a slower renal function deterioration rate. Furthermore, literature review found mutations in different domains of APOE (within or out of the LDL receptor binding region) led to different serum apoE levels. These findings may help us to understand the pathogenic mechanism of LPG, and further indicate that the treatment of proteinuria might slow down renal function progression in these patients.

## CONFLICT OF INTEREST

The authors declare that they have no other relevant financial interests.

## AUTHOR CONTRIBUTION

JX, MY, and QW were involved in the conception and design of the study and writing of the manuscript. XP, SY, JX, and XY were involved in the laboratory experiments and data interpretation. YL, YJ, and CZ were involved in genetic counseling. NC, XL, HR, and XP were involved in patient evaluations. HH revised the manuscript. All authors read and approved the final version of the manuscript.

## Supporting information

Figure S1

Table S1–S2

## Data Availability

The data that support the findings of this study are available on request from the corresponding author. The data are not publicly available due to privacy or ethical restrictions.
