# Supplementary figures and images for: Clinical and genetic analysis of lipoprotein glomerulopathy patients caused by APOE mutations
Source: Mol Genet Genomic Med. 2020 May 22;8(8):e1281. doi: 10.1002/mgg3.1281 (PMC7434605; doi:10.1002/mgg3.1281)

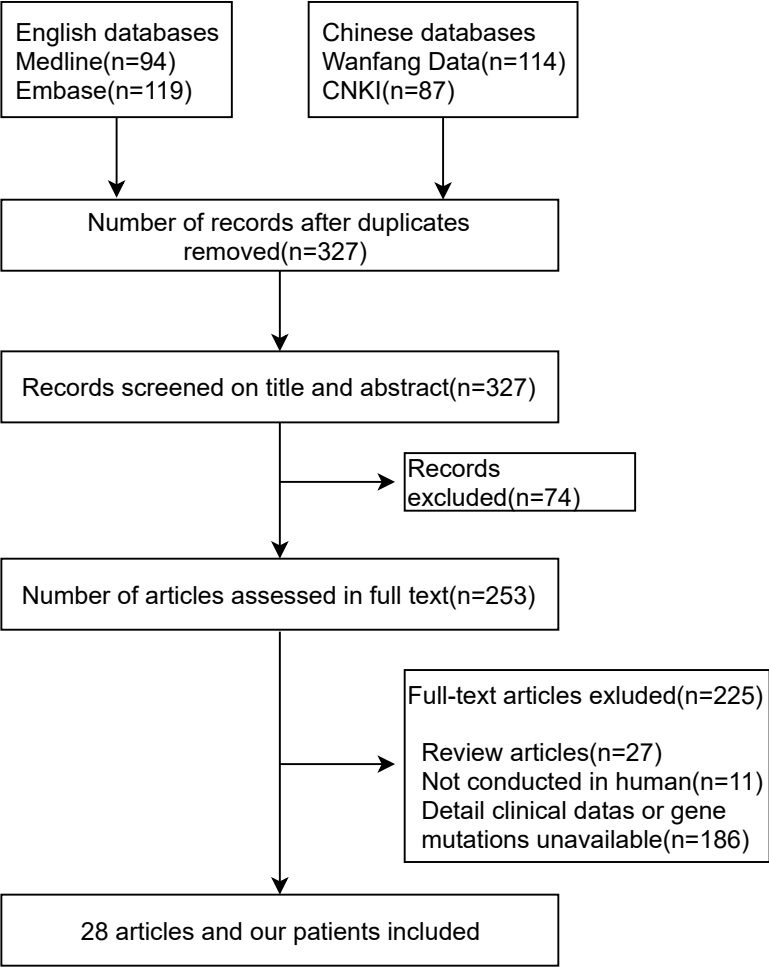

Supplement: Supplementary file 1 — Figure S1 [file MGG3-8-e1281-s001.pdf]
