# Supplementary material for: Clinical and genetic analysis of lipoprotein glomerulopathy patients caused by APOE mutations
Source: Mol Genet Genomic Med. 2020 May 22;8(8):e1281. doi: 10.1002/mgg3.1281 (PMC7434605; doi:10.1002/mgg3.1281)
Supplement: Supplementary file 2 — Table S1–S2 [file MGG3-8-e1281-s002.docx]

**Appendix**

Table S1 The Primer information.

| Exon | Primer sequence (5’→3’) | Annealing temperature (°C) | PCR product size (bp) |
| --- | --- | --- | --- |
| Exon 1 | apoe1-F: GGAGCCCTATAATTGGACAAGT  apoe1-R: TCGTGGAGTCCTGCTATGG | 60 | 341 |
| Exon 2 | apoe2-F: GAGAATGAGGAATGCGAGACTG  apoe2-R: GGAGGTTGAGGTGAGGATGAG | 68 | 447 |
| Exon 3 | apoe3-F: TTGTGGAGCACCTTCTGTGT  apoe3-R: AGCCAGGACGAGTGTGAGA | 64 | 509 |
| Exon 4 | apoe4-1-F: GCATCTGTCTCTGTCTCCTTCT  apoe4-1-R: TGCTCCTTCACCTCGTCCA | 68 | 671 |
|  | Apoe4-2-F: AGAAGCGCCTGGCAGTGTA  Apoe4-2-R: CAGATGCGTGAAACTTGGTGAA | 60 | 581 |

| Table S2 Clinical characteristics of patients with different serum apoE level | | |  |
| --- | --- | --- | --- |
|  | Low apoE group | High apoE group |  |
| n | 23 | 25 |  |
| Age(y) | 34.9±14.0 | 31.1±19.1 |  |
| Male, n (%) | 16(70) | 13(52) |  |
| Positive family history, n (%) | 11(48) | 10(40) |  |
| ln(apoE) (mg/dL) | 1.8±0.4 | 2.8±0.4* |  |
| LDL RBR, n (%) | 8(35) | 21(84)* |  |
| MAP (mmHg) | 101.7±15.6 | 106.8±15.1 |  |
| ln (Upro) (g/dL) | 0.8±1.2 | 1.0±0.9 |  |
| eGFR (mL/min per 1.73 m^2^) | 95.1±28.8 | 95.2±36.6 |  |
| ln (TG) (mmol/L) | 0.9±0.6 | 1.1±0.5 |  |
| ln (TC) (mmol/L) | 1.5±0.5 | 1.9±0.4* |  |
| Low apoE group: LPG patients with apoE <11.2mg/dL; High apoE group: LPG patients with apoE ≥11.2mg/dL; LDL RBG: mutations in low-density lipoprotein receptor binding region; MAP: mean arterial pressure; Upro: urine protein; eGFR: estimated glomerular filtration rate using EPI; TG: triglyceride; TC: total cholesterol.  * compared with low apoE group, p<0.05 | | | |
